# Effects of Acute and Developmental Exposure to Bisphenol S on Chinese Medaka (*Oryzias sinensis*)

**DOI:** 10.3390/jox14020027

**Published:** 2024-03-22

**Authors:** Bingying Li, Yongsi Huang, Duan Pi, Xiang Li, Yafen Guo, Zhiying Liang, Xiaohong Song, Junjie Wang, Xuegeng Wang

**Affiliations:** 1 Institute of Modern Aquaculture Science and Engineering, Guangdong-Macao Joint Laboratory for Aquaculture Breeding Development and Innovation, College of Life Sciences, South China Normal University, Guangzhou 510631, China; 20202531009@m.scnu.edu.cn (B.L.); 20202521021@m.scnu.edu.cn (Y.H.); 20201132026@m.scnu.edu.cn (D.P.); 20202521099@m.scnu.edu.cn (Y.G.); 20202521014@m.scnu.edu.cn (Z.L.); 2 Guangxi Key Laboratory of Environmental Pollution Control Theory and Technology, Guilin University of Technology, Guilin 541000, China; songxh@glut.edu.cn

**Keywords:** BPS, Chinese medaka, developmental exposure, transcriptome

## Abstract

Bisphenol S (BPS), one of the substitutes for bisphenol A (BPA), is widely used in various commodities. The BPS concentrations in surface water have gradually increased in recent years, making it a predominant bisphenol analogue in the aquatic environment and raising concerns about its health and ecological effects on aquatic organisms. For this study, we conducted a 96 h acute toxicity test and a 15-day developmental exposure test to assess the adverse effects of BPS exposure in Chinese medaka (*Oryzias sinensis*), a new local aquatic animal model. The results indicate that the acute exposure of Chinese medaka embryos to BPS led to relatively low toxicity. However, developmental exposure to BPS was found to cause developmental abnormalities, such as decreased hatching rate and body length, at 15 dpf. A transcriptome analysis showed that exposure to different concentrations of bisphenol S often induced different reactions. In summary, environmental concentrations of BPS can have adverse effects on the hatching and physical development of Chinese medaka, and further attention needs to be paid to the potential toxicity of environmental BPS.

## 1. Introduction

Bisphenol A (BPA) is an essential synthetic chemical used in the production of polycarbonate plastics and epoxy resins [1,2]. Measurable concentrations of BPA have been detected in the environmental media around the world [3,4,5,6,7], and exposure to BPA is almost unavoidable [8,9]. BPA is a classic endocrine-disrupting chemical (EDC), which has been demonstrated to have adverse impacts on male reproduction in vertebrates [10,11], prostate development in mammals [12], osmoregulation in fish [13] and may induce obesity [14], dysplasia [15], and cardiovascular diseases [16]. Due to its serious adverse effects, the use of BPA has been banned in many countries and regions. Bisphenol S (BPS) has been gradually developed as a substitute for BPA in polycarbonate plastics and epoxy resins [17,18]. With its growing usage, BPS has been detected in human biological, food, and environmental samples [3,19,20,21,22].

There have been reports of the growing occurrence of BPS in surface waters around the world. Yamazaki et al. reported that the concentrations of BPS in surface water were up to 8.7 ng/L in Japan, 42 ng/L in Korea, 135 ng/L in China, and 7200 ng/L in India [23]. Jin et al. measured the BPS concentration in surface water samples and found that it ranged from 0.22 to 52 ng/L in the Liaohe River, 0.61 to 46 ng/L in the Hunhe River, and 0.28 to 67 ng/L in Taihu Lake [24]. In 2022, the concentrations of BPS in the San Francisco Bay were reported to be up to 120 ng/L [25]. In Europe, the concentrations of BPS were reported to be up to 35.2 ng/L in Slovenia and Croatia [26], 8.23 ng/L in Romania [27], 1584 ng/L in Poland [28], and 306 ng/L in England [29]. In 2017, two studies reported the BPS concentrations in water samples collected from Taihu Lake, which ranged from 4.1 to 160 ng/L in samples collected in November 2016 [30] and 4.5 to 1600 ng/L in samples collected in April 2016 [31]. These studies, especially those in the same location, have shown dramatic increases in BPS concentrations in aquatic environments, drawing attention to the health and ecological effects of environmental BPS exposure on aquatic species [32].

In previous studies, researchers found that BPS has the same order of magnitude of endocrine-disrupting effects as BPA [33]. In addition, studies have shown that BPS exposure may induce obesity [34] and has anti-androgenic properties [35]. In zebrafish embryos and larvae, BPS impacts the reproductive neuroendocrine system during development [36]. Compared with BPA, the current research on the health hazards or effects of BPS on aquatic animals remains limited. It is necessary to determine the adverse impacts of BPS exposure on aquatic animals and its underlying mechanisms.

Small teleost fish are often used as animal models for aquatic toxicology, especially the Japanese medaka (*Oryzias latipes*) and zebrafish (*Danio rerio*) [37,38,39]. These fish have multiple advantages, such as a small size, short generation time, frequent spawning characteristics, complete genome sequences [40,41], and epigenetic reprogramming information [42,43]. However, the responses of zebrafish and Japanese medaka exposed to pollutants differ significantly. For instance, the LC-96 of zebrafish embryos exposed to bisphenol F (BPF)—another substitute for BPA—was 7.40 mg/L [44]. In contrast, the LC-96 of medaka embryos exposed to BPF was approximately 120 mg/L (unpublished data from our lab), approximately 16 times higher than that of zebrafish. Furthermore, the inbred laboratory strains of zebrafish and Japanese medaka do not inhabit wild surface waters, and therefore, it is necessary to include more local aquatic animal models to assess the risk of exposure to environmental pollutants.

Chinese medaka (*Oryzias sinensis*) is related to the Japanese medaka and is found in most parts of East Asia [45]. It has the similar advantages of small individuals, easy feeding and management, a short generation time, and frequent spawning. Previous studies have reported that the Chinese medaka’s toxicological responses to pollutants are similar to that of the Japanese medaka and zebrafish [46,47] but often produces intermediate responses between zebrafish and Japanese medaka [46]. In our previous study, the LC-96 of Chinese medaka embryos exposed to BPF was 87.90 mg/L, which is between those of zebrafish and Japanese medaka [48]. Therefore, the Chinese medaka is an excellent aquatic animal model to fill the gap between zebrafish and Japanese medaka.

In the present study, the hypothesis that exposure to BPS can induce adverse effects on aquatic animals was tested using the Chinese medaka as an animal model. To explore the toxicity of BPS, a 96 h acute exposure and a 15-day developmental exposure to BPS were conducted on Chinese medaka embryos and larvae. The developmental abnormalities were observed, and the transcriptome was analyzed to speculate on the impact of BPS at environmental concentrations on Chinese medaka.

## 2. Materials and Methods

### 2.1. Fish Husbandry

The Chinese medaka were obtained from a local aquarium store and cultured in the laboratory as previously described [48]. All applicable institutional and/or national guidelines for the care and use of animals were followed.

### 2.2. Chemicals

BPS (CAS No. 80-09-1; purity 98%) was purchased from Aladdin (Shanghai, China). DMSO (CAS:67-68-5) was purchased from Sigma-Aldrich (St. Louis, MO, USA). The BPS stock solution was made in DMSO at a 1 g/mL concentration. The stock and exposure BPS solutions were stored in the dark at 4 °C.

### 2.3. Acute Exposure to BPS

A series of BPS concentrations (250, 500, 750, and 1000 mg/L) were prepared for the acute toxic exposure following the equal difference interval method to investigate the acute exposure toxicity of BPS toward Chinese medaka embryos. The highest concentration is close to the reported water solubility (1100 mg/L) [49]. The concentration of DMSO in each group was controlled at 0.1% (*v*/*v*). Each treatment was performed in triplicate.

Newly produced embryos were randomly distributed into each treatment group. For each treatment, 10 embryos were kept in each well of a 6-well culture plate. The exposure solution was renewed daily. The survival rates of the embryos in each treatment group were recorded at 24, 48, 72, and 96 hpf (hours post fertilization), and any dead embryos were removed. The experiment was conducted following the guiding principles of OECD (No. 212) [50].

### 2.4. Developmental Exposure to BPS

Previous research has shown that BPS is ubiquitous in aquatic environments [51]. To explore whether the environmental concentrations of BPS will produce toxic effects on aquatic organisms, a series of BPS concentrations (20, 200, and 2000 ng/L), which cover the environmental concentrations, were used in this study. The concentration of the DMSO in each group was controlled at 0.0001% (*v*/*v*), and a blank control group with water was included. As there is no significant difference and only one differentially expressed genes (DEGs) was found between the solvent and blank control groups in a parallel study [48], only the blank control group was used in the following analysis. Each treatment group had 6 replicates. The embryos were randomly divided into the wells of 6-well culture plates and exposed to different BPS concentrations from the blastula stage until 15 dpf (days post fertilization). Each group in a single well contained 10 embryos. The exposure solution was replaced every two days. The plates were all placed in an environmental chamber at 26.8 °C with a 14/10 h light/dark cycle. The experiment was conducted following the guiding principles of OECD (No. 210) [52].

To measure the concentrations of BPS, 500 mL of the exposure solution was collected for each sample. The pH of the BPS sample was adjusted to 5 ± 0.02 using 0.1 mol/L diluted hydrochloric acid. Subsequently, solid-phase extraction was employed to extract and purify the treated BPS samples. The quantitative analysis phase utilized high-performance liquid chromatography tandem mass spectrometry (UHPLC-MS/MS). The chromatographic conditions involved mobile phase A (1 mmol/L ammonium fluoride solution), mobile phase B as pure acetonitrile, and gradient elution for effective separation. In negative ion source mode, mass spectrometry conditions specified a precursor ion (*m*/*z*) of 249/155.5 for monitoring BPS, and a product ion (*m*/*z*) of 107.9 for quantitative analysis. The cone voltage was set at 24, and the collision energy was set at 16/16, generating fragment ions to enhance quantitative accuracy. Ensuring a linear relationship, a standard curve was established for quality control and assurance. A correlation coefficient R^2^ ≥ 0.997 indicates an excellent fit of the standard curve, meeting the requirements for a high-quality analysis and ensuring the reliable and accurate quantitative analysis of BPS.

### 2.5. Morphology Observation and Sample Collection

During the developmental exposure test, the hatching rate, survival rate, heartbeat rate, and blood circulation of larvae were examined under a microscope and recorded daily. At 15 dpf, the body length, heartbeats, and various abnormal phenotypes, including pericardial edema (ce), spinal curvature, enlarged yolk sac (cv), and decreased head–trunk angle (HTA↓) were recorded. After the measurements were completed, whole larvae samples were transferred into the Trizol reagent (Invitrogen, Waltham, MA, USA) and then stored at −80 °C for the following analysis.

### 2.6. Transcriptomic Analysis

#### 2.6.1. Library Construction and Sequencing

The libraries were prepared as previously described [48]. In brief, the total RNA was extracted using Trizol reagent according to the manufacturer’s instructions. RNA quality was assessed with an Agilent 2100 Bioanalyzer (Agilent Technologies, Santa Clara, CA, USA) and agarose gel electrophoresis. mRNA was enriched using Oligo(dT) beads, followed by fragmentation and reverse transcription with random primers. Then, the cDNA fragments were purified and end-repaired. The A base was added to the end of the fragments. Then, the fragments were ligated to Illumina sequencing adapters. After size selection through agarose gel electrophoresis, the ligation products were PCR amplified and sequenced using an Illumina Novaseq 6000.

#### 2.6.2. Data Processing

The raw sequencing reads were filtered using fastp [53] (version 0.18.0) and mapped to the Chinese medaka reference genome (ASM858656v1). The unique mapped reads from each sample were assembled with StringTie (v1.3.1) [54,55]. To quantify the expression abundance and variations, an FPKM (fragment per kilobase of transcript per million mapped reads) value was calculated for each transcription region using RSEM software (v1.3.3) [56]. To obtain the differentially expressed genes (DEGs), the unique mapped reads were then analyzed using DESeq2 (v1.36.0) [57]. A fold change (FC) ≥ 2 and false discovery rate (FDR) < 0.05 were considered significant.

#### 2.6.3. GO and KEGG Enrichment Analyses

To perform the gene ontology (GO) analysis [58], the DEGs were mapped to GO terms in the gene ontology database (http://www.geneontology.org; accessed on 3 February 2023), and the significantly enriched GO terms were defined through the hypergeometric test and an adjusted *p* < 0.05. KEGG (Kyoto Encyclopedia of Genes and Genomes) [59] enrichment analysis was performed to further understand the biological functions of the DEGs and their interaction with each other in certain biological functions. Pathways with an adjusted *p* < 0.05 were defined as significantly enriched.

### 2.7. Statistical Analysis

The data are presented as the mean ± SEM (standard error of the mean). The differences among treatment groups were determined with one-way ANOVA, and multiple comparisons were performed using Tukey’s test. The *t*-test was also performed, if necessary. *p* < 0.05 was considered statistically significant.

## 3. Results

### 3.1. Acute BPS Toxicity Test on Embryos

In the 96 h acute toxicity test, embryonic lethality rarely occurred. As the exposure concentration of BPS increased and approached its solubility limit in water, the survival rate only slightly decreased (Figure S1). There were no significant differences among the groups using one-way ANOVA, indicating that high concentrations of BPS have a relatively low toxicity in Chinese medaka.

### 3.2. Developmental BPS Exposure Test

**Figure 1:**
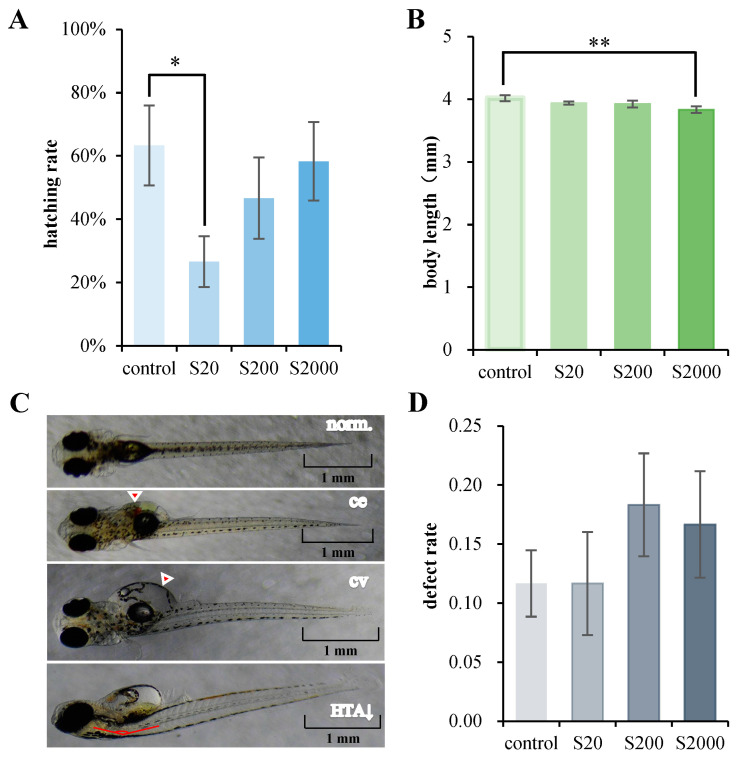
Developmental BPS exposure induced abnormalities at 15 dpf. (**A**) Hatching rate of larvae; (**B**) Body length of larvae; (**C**) Illustrations of normal fry (norm.) and fry with pericardial edema (ce), enlarged yolk sac (cv), and decreased head–trunk angle (HTA↓); (**D**) Defect rate of larvae with multiple abnormalities. Data are represented as mean ± SEM. * *p* < 0.05; ** *p* < 0.01.

In the 15-day developmental exposure experiment, the actual mean measured concentrations of BPS in the 20, 200, and 2000 ng/L treatment groups were 16.6 ± 0.3, 167.3 ± 11.2, and 1735.3 ± 54.2 ng/L, respectively. In the following, these three BPS treatment groups are expressed as nominal concentrations and are referred to as S20, S200, and S2000, respectively. All embryos survived during the developmental exposure, indicating that the environmental concentration of BPS (20, 200, and 2000 ng/L) generally does not cause fatal effects in the early stages of Chinese medaka. We recorded and analyzed the growth parameters of juvenile fish, including hatching rate, heartbeat, and body length. The hatching rate of the S20 group significantly decreased (Figure 1A), and the body length of the S2000 group significantly decreased compared to the control (Figure 1B). There were no significant differences in heartbeat (Figure S2D).

Multiple developmental abnormalities were examined (Figure 1C). No significant differences were observed in pericardial edema (Figure S2A), enlarged yolk sac (Figure S2B), and decreased head–trunk angle (Figure S2C). Different degrees of increases were found for several abnormalities, demonstrating that BPS at environmental concentrations may cause health defects in Chinese medaka larvae (Figure 1D).

### 3.3. RNA Sequencing and Transcriptome Assembly

To further illustrate the underlying mechanisms through which the BPS exposure caused adverse impacts, a transcriptome analysis was performed. After performing quality control, 57,363,422 to 75,195,674 high-quality clean reads were generated with RNA-seq for each sample, and the unique mapping ratio ranged from 74.34% to 75.93% (Table S1).

**Figure 2:**
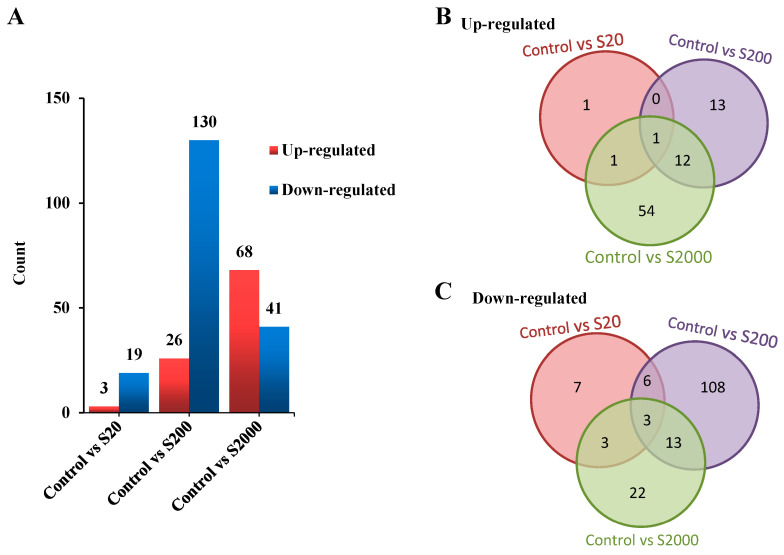
Statistics for DEGs. (**A**) The numbers of up-regulated and down-regulated DEGs in the BPS treatment groups; (**B**) Venn diagram showing the shared up-regulated DEGs among the groups; (**C**) Venn diagram showing the shared down-regulated DEGs among the groups (S20, S200, S2000 represent the 20 ng/L BPS, 200 ng/L BPS, 2000 ng/L BPS treatments, respectively).

The transcriptomes were assembled, and the DEGs were identified. The results showed that 22, 156, and 109 DEGs were identified between the control versus the S20, S200, and S2000 groups, respectively (Table S2). Briefly, 3 genes were up-regulated, and 19 genes were down-regulated in the control vs. S20; a total of 26 genes were up-regulated, and 130 genes were down-regulated in the control vs. S200; and 68 genes were up-regulated, and 41 genes were down-regulated in the control vs. S2000 (Figure 2A).

As shown in Figure 2B,C, one gene was up-regulated (Figure 2B), and nine genes were down-regulated (Figure 2C) in both the control vs. S20 and S200 comparisons. Two genes were up-regulated (Figure 2B), and six genes were down-regulated (Figure 2C) in both the control vs. S20 and S2000 comparisons. A total of 13 genes were up-regulated, (Figure 2B) and 16 genes were down-regulated (Figure 2C) in both the control vs. S200 and S2000 comparisons.

### 3.4. Gene Ontology Analysis

To analyze the molecular level harmful effects of BPS on Chinese medaka embryos, gene ontology enrichment was performed (Table S3).

**Figure 3:**
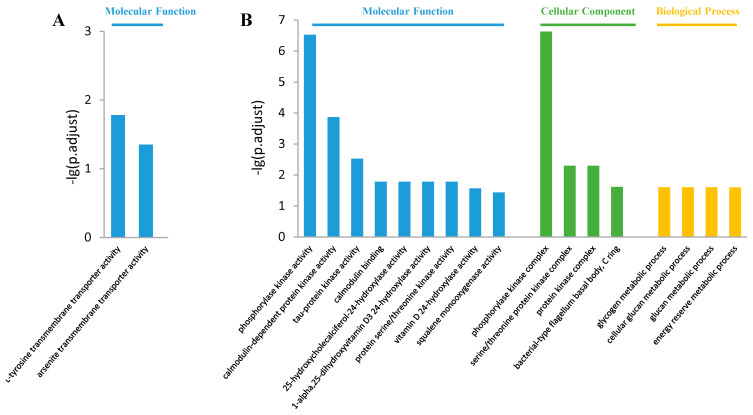
Gene ontology term enrichment in the control vs. S20 comparison. (**A**) Top enriched GO terms among up-regulated DEGs; (**B**) Top 10 enriched GO terms among down-regulated DEGs.

The most enriched GO terms in the control vs. S20 comparison are shown in Figure 3. In the molecular function class, the up-regulated DEGs were enriched in “L-tyrosine transmembrane transporter activity” and “arsenite transmembrane transporter activity”, while the down-regulated DEGs were enriched in “phosphorylase kinase activity”, “calmodulin-dependent protein kinase activity”, “tau-protein kinase activity”, “protein serine/threonine kinase activity”, “calmodulin binding”, “vitamin D 24-hydroxylase activity”, and “squalene monooxygenase activity” items (Figure 3A,B). In the cellular component class, only the down-regulated DEGs were enriched in “phosphorylase kinase complex”, “serine/threonine protein kinase complex”, “protein kinase complex”, and “bacterial-type flagellum basal body, C ring” items (Figure 3B). In the biological process class, only the down-regulated DEGs were enriched in “glucan metabolic process”, “glycogen metabolic process”, “cellular glucan metabolic process”, and “energy reserve metabolic process” items (Figure 3B).

**Figure 4:**
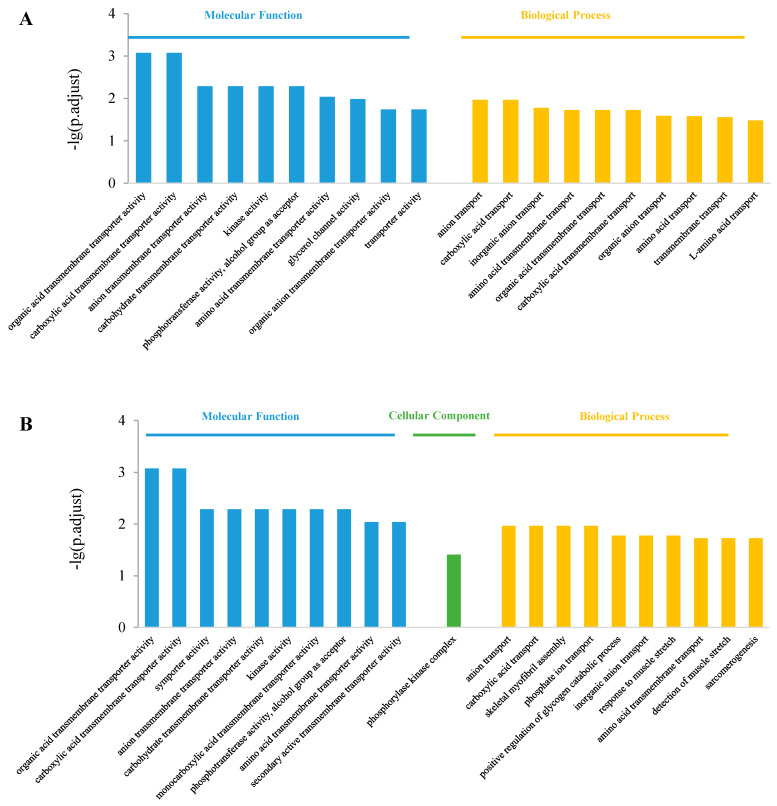
Gene ontology enrichment in the control vs. S200 comparison. (**A**) Top 10 enriched GO terms among up-regulated DEGs; (**B**) Top 10 enriched GO terms among down-regulated DEGs.

The top 10 enriched GO terms in the control vs. S200 comparison are shown in Figure 4. In the molecular function class, both up-regulated and down-regulated DEGs were enriched in “organic acid transmembrane transporter activity”, “phosphotransferase activity, alcohol group as acceptor”, “carboxylic acid transmembrane transporter activity”, “kinase activity”, “carbohydrate transmembrane transporter activity”, and “amino acid transmembrane transporter activity” items (Figure 4A,B). In the cellular component class, only down-regulate DEGs were enriched in the “phosphorylase kinase complex” item (Figure 4B). In the biological process class, both up- and down-regulated DEGs were enriched in “anion transport”, “carboxylic acid transport”, “inorganic anion transport” (Figure 4A,B). The up-regulated DEGs were also enriched in the “organic acid transmembrane transport” and “carboxylic acid transmembrane transport” (Figure 4A). While the down-regulated DEGs were enriched in the “skeletal myofibril assembly”, “response to muscle stretch”, “detection of muscle stretch”, and “sarcomerogenesis” items, which are related to movement (Figure 4B).

**Figure 5:**
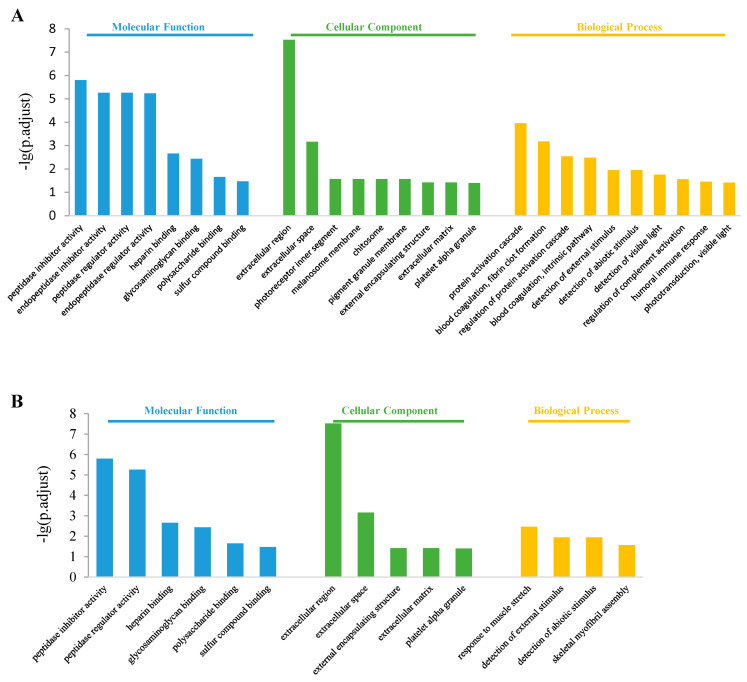
Gene ontology enrichment in the control vs. S2000 comparison. (**A**) Top 10 enriched GO terms among up-regulated DEGs; (**B**) Top 10 enriched GO terms among down-regulated DEGs.

The top 10 enriched GO terms in the control vs. S2000 comparison are shown in Figure 5. In the molecular function class, both up- and down-regulated DEGs were enriched in “peptidase regulator activity”, “peptidase inhibitor activity”, “heparin binding”, “glycosaminoglycan binding”, “polysaccharide binding”, and “sulfur compound binding” items (Figure 5A,B). The up-regulated DEGs were also enriched in the “endopeptidase regulator activity” and “endopeptidase inhibitor activity” items (Figure 5A). In the cellular component class, the DEGs were enriched in “extracellular region”, “extracellular space”, “pigment granule membrane”, and “external encapsulating structure” items (Figure 5A,B). The up-regulated DEGs were also enriched in the “chitosome” and “melanosome membrane” items (Figure 5A). In the biological process class, the up-regulated DEGs were enriched in “protein activation cascade”, “regulation of protein activation cascade”, “blood coagulation, fibrin clot formation”, “detection of external stimulus”, “detection of abiotic stimulus”, and “detection of visible light” (Figure 5A), while the down-regulated DEGs were enriched in “response to muscle stretch”, “detection of abiotic stimulus”, “detection of external stimulus”, and “skeletal myofibril assembly” items (Figure 5B).

### 3.5. KEGG Analysis

**Figure 6:**
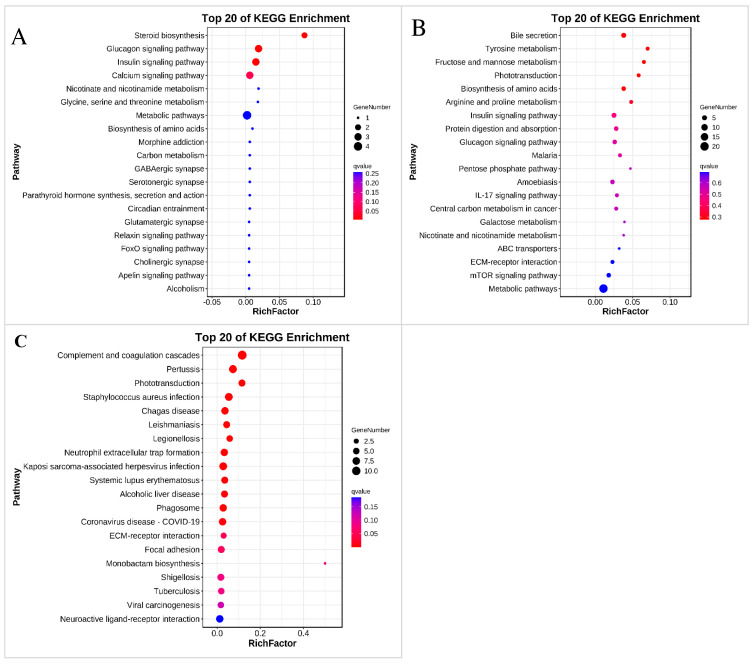
KEGG enrichment. (**A**) Top 20 enriched pathways of the DEGs in the control vs. S20 comparison; (**B**) Top 20 enriched pathways of the DEGs in the control vs. S200 comparison; (**C**) Top 20 enriched pathways of the DEGs in the control vs. S2000 comparison.

In the comparison of the S20 and control groups, the DEGs were annotated into 12 functional categories and were significantly enriched in “Steroid biosynthesis”, “Glucagon signaling pathway”, and “Insulin signaling pathway” (Figure 6A and Table S4). In the comparison of the S200 and control groups, the DEGs were annotated into 34 functional categories, but no pathway was statistically significantly enriched (Figure 6B and Table S4).

In the comparison of the S2000 and control groups, the DEGs were annotated into 36 functional categories and were significantly enriched in 14 KEGG pathways, including “Complement and coagulation cascades”, “Pertussis”, “Legionellosis”, “Kaposi sarcoma-associated herpesvirus infection”, “Coronavirus disease—COVID-19”, “Phagosome”, “Neutrophil extracellular trap formation”, “Chagas disease”, “Alcoholic liver disease”, “Systemic lupus erythematosus”, “Staphylococcus aureus infection”, “Leishmaniasis”, “ECM-receptor interaction”, “Phototransduction”, and “Insulin signaling pathway” (Figure 6C and Table S4).

## 4. Discussion

With the increasing use of BPS, more research has been performed, which has shown that BPS has various potential toxicities in various animal models [60]. Using Chinese medaka as an animal model, the present study assessed the acute toxicity of BPS exposure on embryos and the developmental toxicity of BPS at environmental concentrations on embryos and larvae. The 96 h acute exposure showed that BPS was not lethal up to 1000 mg/L for Chinese medaka embryos. In a previous study, for a 96 h acute exposure, the LC50 of bisphenol F (BPF) was 87.90 mg/L for Chinese medaka embryos [48], suggesting that BPS is less toxic than BPF. In cell models, researchers found that the LC50 of BPS on fish primary macrophages was 39.1 mg/L and 29.7 mg/L after a 6 h or 12 h exposure, respectively [61]. In zebrafish, the 96 h LC50 of BPS was reported to be 199 mg/L [62]. Compared with other experimental animals, Chinese medaka seems less sensitive to BPS in the acute toxicity tests.

In the developmental exposure experiment, the physical features of the larvae in the S2000 groups showed that the body length of larvae was significantly decreased compared to the control group. In zebrafish, developmental exposure to 100 μg/L BPS significantly reduced the body length at 5 dpf [63], indicating the conserved toxicological effects of BPS exposure on growth in teleosts. The defect rates of all embryos in the S200 and S2000 groups increased to different extents, which corresponds to the number of enriched DEGs in these two comparisons. These results indicate that the concentration of BPS in the developmental exposure dose was harmful to Chinese medaka embryos.

Among the research on the environmental concentration of BPS, it was found to reach 7.2 × 10^3^ ng/L in the Adyar River in India [23], much higher than the concentrations used for the developmental exposure in this study. In the Taihu Lake in China, the BPS concentration also reached as high as 1.6 × 10^3^ ng/L, which was close to the highest concentration used in this study, suggesting a high ecotoxicological risk. Furthermore, BPS was frequently detected with other bisphenol analogues in almost all mediums [21,24,25,26,30,32]. It is crucial to examine the adverse effects induced by joint exposure to multiple bisphenol analogues in the future.

Environmental disturbances during embryogenesis can cause subtle functional changes, altering gene expression, physiology, and metabolism [30]. Due to its endocrine-disrupting characteristics, the effects induced by exposure to bisphenol analogues are usually not monotonous. In the present study, the comparison of the control vs. S200 group had the most DEGs. According to the GO analysis, the DEGs associated with the enriched GO terms in the control vs. S20 comparison were mostly down-regulated, while those in the control vs. S2000 comparison were mostly up-regulated. These results indicate that BPS employs various molecular mechanisms in its toxicity at different concentrations.

Intriguingly, the “regulation of complement activation” and “humoral immune response” terms were highly enriched in the control vs. S2000 comparison. The S2000 group also had DEGs enriched in the immune-related pathways, such as “Complement and coagulation cascades” and “Neutrophil extracellular trap formation”. Both results indicate that developmental exposure to 2000 ng/L BPS significantly affected the gene expression in the immune system. Similarly, exposure to 10 μg/L BPS significantly altered the expression of genes involved in the innate immune system in zebrafish, including the *tnf-α* and *ifn* genes [64]. The immune system can actively respond to various environmental stresses, which is crucial to organisms’ survival. More investigations are needed to uncover the mechanisms involved in the immunotoxicity of BPS.

Moreover, the GO terms involving glycogen and lipid metabolism and transformation processes were frequently enriched, suggesting that BPS could hinder the energy conversion and absorption processes in organisms. This analysis was consistent with the results of the embryos with enlarged yolk sacs in the BPS developmental exposure experiment. This phenomenon is also reflected in other instances where BPS was demonstrated to interfere with yolk lipid consumption in zebrafish embryos [65]. This result indicates that BPS exposure may cause harmful effects on energy metabolism.

Recently, a cross-sectional study in the USA, which involved 1521 participants aged 20 years or older, found that urinary BPS concentrations were higher in obese participants [66]. Other studies have demonstrated that BPS can bind to nuclear receptors in fatty tissues, contributing to the development of obesity [67]. We also found a significant enrichment of the “Kaposi sarcoma-associated herpesvirus infection”, “Chagas disease”, “Alcoholic liver disease”, “Systemic lupus erythematosus”, “Staphylococcus aureus infection”, “Leishmaniasis”, “Pertussis”, and “Legionellosis” KEGG pathways in Chinese medaka embryos exposed to environmental BPS. These pathways are associated with disease. The enrichment of these pathways maybe because BPS increases the incidence of related diseases.

Besides the potential adverse health effects on humans, the pollution of aquatic ecosystems with EDCs has multiple impacts on non-human animals. Aquatic organisms are also an essential food source for humans, creating the complex relationships between EDCs and people, animals, plants, and the environment. Hence, it is necessary to consider the consequences of EDCs, such as BPS, from multiple dimensions with One Health thinking [68,69]. Furthermore, there are no two identical environments in the world, and so, it is critical to include more local animal models besides laboratory animal models to investigate the impacts of pollutants. In the present and previous studies, Chinese medaka has been proven to be an ideal fish model to fill the gap between laboratory and field work with its multiple advantages [45,47,48,70,71,72].

## 5. Conclusions

This study revealed that BPS had low acute toxicity on Chinese medaka embryos during a 96 h acute toxic exposure. However, the developmental toxicity of BPS on Chinese medaka embryos and larvae induced several developmental abnormalities, indicating that environmental concentrations of BPS may pose an ecological risk for aquatic organisms. GO and KEGG pathway analyses suggested that BPS exposure at environmental concentrations can affect the movement, metabolism, immune system, and disease-related genes.

## Data Availability

The raw sequence data reported in this paper have been deposited in the Genome Sequence Archive (GSA) in National Genomics Data Center under accession number CRA012060, which is publicly accessible at https://ngdc.cncb.ac.cn/gsa (accessed on 4 December 2023).

## Supplementary Materials

The following supporting information can be downloaded at: https://www.mdpi.com/article/10.3390/jox14020027/s1, Table S1: Data statistics of clean data; Table S2: DEGs between every two different groups; Table S3: Enriched GO terms: Table S4: The top 20 KEGG pathways; Figure S1: Results of the acute toxic experiment. Survival rates of Chinese medaka embryos exposed to 0 (control), 250, 500, 750, 1000 mg/L BPS for 96 h; Figure S2: The growth parameters of the 15 dpf larvae after BPS exposure. (A): The rate of Chinese medaka with pericardial edema (ce); (B): The rate of Chinese medaka with enlarged yolk sac (cv); (C): The rate of Chinese medaka with decreased head-trunk angle (HTA↓); (D): Heart beats (bpm, beats per minute) of the larvas.
