# Supplementary material for: Effects of Acute and Developmental Exposure to Bisphenol S on Chinese Medaka (Oryzias sinensis)
Source: J Xenobiot. 2024 Mar 22;14(2):452–66. doi: 10.3390/jox14020027 (PMC10961820; doi:10.3390/jox14020027)
Supplement: Supplementary file 1 [file jox-14-00027-s001.zip › Supplementary Information 20231205.pdf]

Supplementary Information

Effects of Acute and Developmental Exposure to Bisphenol S on Chinese Medaka (*Oryzias sinensis*)

Bingying Li [1,†], Yongsi Huang [1,†], Duan Pi [1], Xiang Li [1], Yafen Guo [1], Zhiying Liang [1], Xiaohong Song [2], Junjie Wang [1,*] and Xuegeng Wang [1,*]

[1] Institute of Modern Aquaculture Science and Engineering, Guangdong-Macao Joint Laboratory for Aquaculture Breeding Development and Innovation, College of Life Sciences, South China Normal University, Guangzhou 510631, China; 20202531009@m.scnu.edu.cn (B.L.); 20202521021@m.scnu.edu.cn (Y.H.); 20201132026@m.scnu.edu.cn (D.P.); 20212521004@m.scnu.edu.cn (X.L.); 20202521099@m.scnu.edu.cn (Y.G.); 20202521014@m.scnu.edu.cn (Z.L.)

[2] Guangxi Key Laboratory of Environmental Pollution Control Theory and Technology, Guilin University of Technology, Guilin 541000, China; songxh@glut.edu.cn

* Correspondence: 20185210@m.scnu.edu.cn (J.W.); wangxuegeng@scnu.edu.cn (X.W.)

† These authors contributed equally to this work.

Supplemental Figures

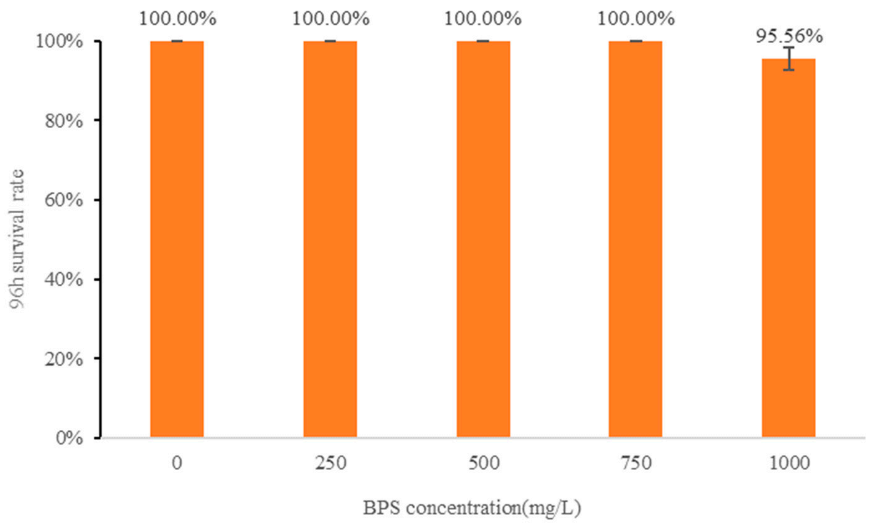

Suppl Figure S1: Results of the acute toxic experiment. Survival rates of Chinese medaka embryos exposed to 0 (control), 250, 500, 750, 1000 mg/L BPS for 96 h.

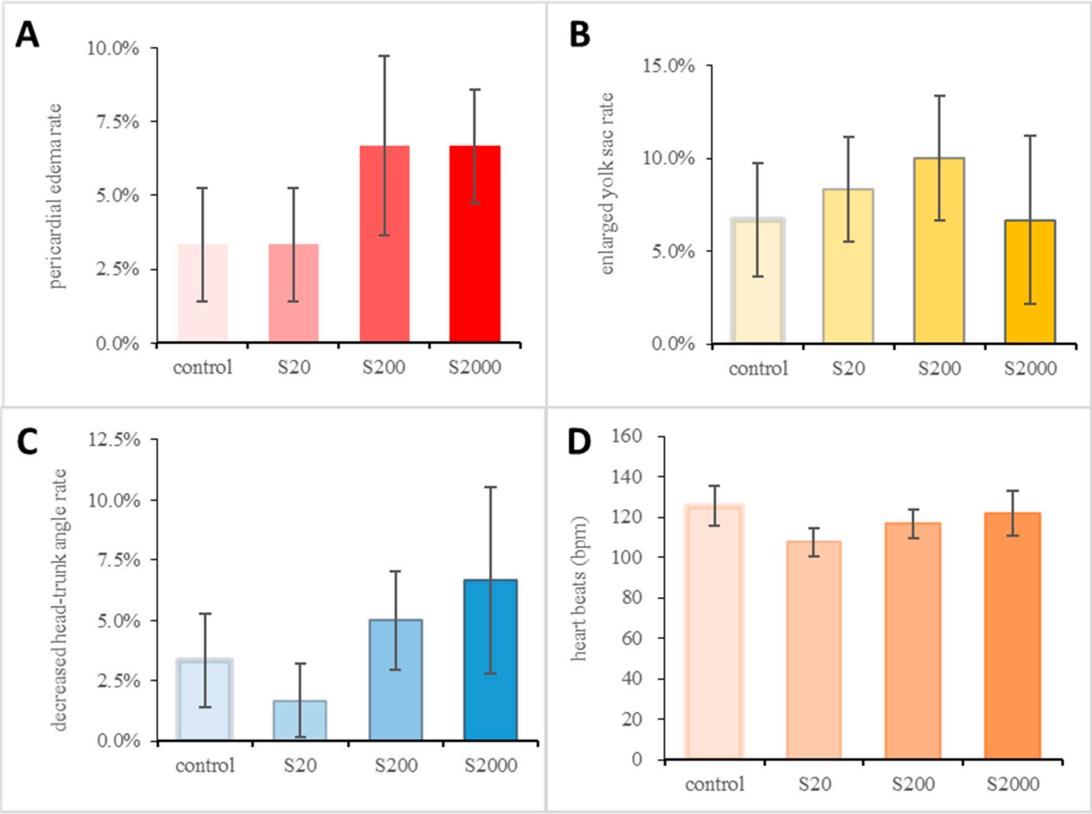

Suppl Figure S2: The growth parameters of the 15 dpf larvae after BPS exposure. (A): The rate of Chinese medaka with pericardial edema (ce); (B): The rate of Chinese medaka with enlarged yolk sac (cv); (C): The rate of Chinese medaka with decreased head-trunk angle (HTA↓); (D): Heart beats (bpm, beats per minute) of the larvas.
